# Myokine BDNF highly expressed in Type I fibers inhibits the differentiation of myotubes into Type II fibers

**DOI:** 10.1007/s11033-024-10044-3

**Published:** 2024-11-12

**Authors:** Teng Hu, Yasuro Furuichi, Yasuko Manabe, Kenichiro Yamada, Kengo Katakura, Yuna Aoki, Kun Tang, Takaomi Sakai, Nobuharu L. Fujii

**Affiliations:** 1 Department of Health Promotion Sciences, Graduate School of Human Health Sciences, Tokyo Metropolitan University, 1-1 Minami-Osawa, Hachioji, Tokyo 192-0397 Japan; 2 Department of Biological Sciences, Graduate School of Science, Tokyo Metropolitan University, 1-1 Minami-Osawa, Hachioji, Tokyo 192-0397 Japan

**Keywords:** BDNF, Myokine, Muscle fiber type, Myotube

## Abstract

**Background:**

Myofibers are broadly classified as slow-twitch (Type I) and fast-twitch (Type II) fibers. These two types of myofibers coexist within the same skeletal muscle tissue, determining the contractile and metabolic properties of skeletal muscle tissue by fiber type distribution.

**Methods and results:**

By examining each fiber type separately, we confirmed that brain-derived neurotrophic factor (BDNF) gene is highly expressed in Type I fibers. When exposed to BDNF, primary myotubes exhibited reduced expression of Myosin Heavy Chain (MyHC) II, a marker protein characteristic of Type II fibers. BDNF overexpression in regenerating muscle tissue led to a decrease in the distribution of Type IIA fibers.

**Conclusions:**

We suggest that BDNF highly expressed in Type I fibers downregulates MyHC II expression in myotubes, eventually inhibiting Type IIA fiber generation.

**Supplementary Information:**

The online version contains supplementary material available at 10.1007/s11033-024-10044-3.

## Introduction

Skeletal muscle is a highly organized tissue composed of bundles of multinucleated muscle fibers called myofibers. Muscle fibers are broadly classified as slow-twitch (Type I) and fast-twitch (Type II) fibers [1]. While the fiber-type distribution is traditionally believed to be genetically determined, studies have reported that adult muscle fiber type distribution can be altered by various physiological factors [2–4].

Myokines, bioactive molecules released by muscle cells, have been considered to act on distal organs in endocrine manner [5, 11]. They also act on cells adjacent to myofibers like satellite cells in paracrine manner [6]. Myokine R-spondin 3 is specifically expressed in Type I fibers and guides the differentiation of myoblasts into Type I fibers by upregulating myosin heavy chain (MyHC) I expression [7]. We propose that muscle fiber type specific myokines play a critical role in fiber type determination during myogenesis.

Myogenesis relies on myogenic stem cells called satellite cells, which proliferate as myoblasts upon muscle injury and subsequently differentiate into myotubes and terminally mature myofibers. Given this, muscle fibers of a specific type could secrete their unique myokines to guide newly formed muscle fibers to differentiate into their own type. This mechanism is potentially related to the preservation of the specificity of the muscle fiber population during regeneration.

To examine fiber type specific myokines, transgenic mice expressing cyan fluorescent protein (CFP) under Myh7 promotor were adapted. Only Type I fibers can yield a cyan fluorescence, allowing us to separate Type I and Type II fibers from the same muscle tissue. We found that Type I fibers express approximately twice as much brain-derived neurotrophic factor (BDNF) mRNA compared to Type II fibers in the soleus. We speculate that BDNF may be related to Type I fiber determination. However, a report [13] has indicated that BDNF mediates glycolytic fiber type specification in vivo, namely Type II fibers. This contradicts our speculation, as it seems counterintuitive for factors predominantly expressed in Type I fibers to mediate Type II fiber specification.

The aim of this study is to confirm how BDNF regulates muscle fiber type determination during myogenesis. Our finding reveals that BDNF downregulates the expression of Type II fiber-specific protein MyHC II in differentiating myotubes and inhibits Type IIA fibers generation in muscle tissue.

## Materials and methods

### Animal

Myh7-CFP Mice (Stock no. 016922) obtained from the Jackson Laboratory (ME, USA.), were used for BDNF gene expression analysis and primary cell culture [8]. Wild type C57BL/6 J mice (Sankyo Lab Service, Tokyo, Japan) were used for experiments overexpressing BDNF in skeletal muscle. Male mice between 8 and 12 weeks were used in this study.

### Isolation of single muscle fibers and primary cell culture

We collected muscle fibers for RNA extraction and satellite cell culture following the protocol in our previous study [7]. CFP fluorescence was observed under a fluorescence stereomicroscope (Leica M165 FC), allowing us to identify each muscle fiber as Type I (CFP-positive) or Type II (CFP-negative).

Satellite cells were cultured according to methods in our previous report [15]. In this study, myoblasts were seeded at 5.0 × 10^4^ cells/well in 24-well plates. BDNF (R&D Systems, MN, USA, 248-BDB-010) was added to the differentiation medium at a concentration of 100 ng/mL (7.41 nM) either at the start of differentiation or on day 3 for 72 h. For the experiment focusing on the pharmacological activation of the neurotrophic receptor tyrosine kinase 2 (TrkB), the same experimental protocol was followed, except BDNF was replaced by the TrkB agonist PG003 at a concentration of 20 ng/mL (3.88 nM, provided by PeptiGrowth Corporation, Tokyo, Japan).

### DNA injection into regenerating skeletal muscle and in vivo electroporation

To induce muscle regeneration, 50 µL of 10 µM Cardiotoxin (Funakoshi, Tokyo, Japan) was injected into both sides of the tibialis anterior (TA) muscle of 9-week-old mice. Three days post-injection, either empty pCAGGS vector (provided by Dr. Junichi Miyazaki from Osaka University) or plasmids encoding BDNF (pCMV6 BDNF; Addgene, MA, USA) were diluted in 0.9% NaCl saline to a concentration of 2 μg/μL. Following anesthetization, 25 μL of the DNA solution was injected intramuscularly into the TA along the long axis of the muscle fibers using an insulin syringe. Immediately after injection, an electrode and a pair of stainless-steel needles were inserted into the skeletal muscle, and it was stimulated with eight square-wave electric pulses (200 V/cm) using an electrical pulse generator (Uchida Denshi, Hachioji, Japan). Muscles were dissected and analyzed 11 days after electroporation.

### Immunostaining

To examine myoblast proliferation, we seeded 20 myofibers/well to 24-well plate containing growth medium, either supplemented with 100 ng/mL recombinant BDNF or unsupplemented for 6 days. Myoblasts were then fixed with 4% paraformaldehyde (PFA) and blocked in PBS containing 0.3% Triton X-100 and 10% goat serum for 30 min at room temperature. Proliferative cells were identified by incubation with Ki67 primary antibody (D3B5, Cell Signaling technology; MA, USA, 1:400) overnight followed by staining with Alexa Fluor 594 secondary antibody (Thermo Fisher Scientific, MA, USA, 1:400) for 60 min. Nuclei were labeled by 4′,6-diamidino-2-phenylindole (DAPI). The cells were subsequently observed through a fluorescence microscope BZ-X810 (Keyence, Osaka, Japan).

To examine myoblast differentiation, we treated myoblasts with differentiation medium supplemented with BDNF or left unsupplemented from the start of differentiation for 3 days. Then differentiated myotubes were fixed with 4% PFA and blocked in PBS containing 0.3% Triton X-100 and 5% goat serum for 60 min at room temperature. The cells were incubated with myosin heavy chain primary antibody (MAB4470, R&D Systems, 1:400) at 4 °C overnight. Following incubation with Alexa Fluor 488 secondary antibody for 60 min, the nuclei were counterstained using DAPI.

To examine the effect of BDNF in regenerating muscle, immunohistochemistry was performed based on a previous study [9]. The tibialis anterior (TA) sections were embedded in O.C.T. Compound (Sakura Finetek, Tokyo, Japan) and quickly frozen in 2-methylbutane that had been pre-cooled with liquid nitrogen. Frozen tissue Sects. (10 µm in thickness) were mounted on glass slides. Sections were blocked with M.O.M. Blocking reagent (Vector Laboratories, Newark, CA, USA) and incubated overnight at 4 °C with the following primary antibodies: MyHC I (BA-D5, DSHB, IA, USA.), MyHC IIA (SC-71), MyHC IIB (BF-F3) and laminin (L9393, Sigma, MO, USA.). The sections were incubated with a mixture of second antibodies including Alexa Fluor 350 mouse IgG2b, Alexa Fluor 488 mouse IgG1, Alexa Fluor mouse 555 IgM and Alexa Fluor rat 647 IgG for 1 h at room temperature.

### Conventional PCR and real time quantitative PCR

Total RNA was extracted from the soleus, extensor digitorum longus (EDL), tibialis anterior (TA), brain, and each type of muscle fiber using Trizol reagent (Invitrogen, CA, USA). cDNA was synthesized using the PrimeScript™ 1st strand cDNA Synthesis Kit (Takara, Shiga, Japan) following the manufacturer’s instructions. The presence of BDNF transcript was analyzed by conventional PCR (RT-PCR). PCR was performed using TaKaRa Taq (Takara) with specific primer pairs: BDNF (Fw: ATTAGCGAGTGGGTCACAGC; Rv: TCTTCCCCTTTTAATGGTCAGTGT); GAPDH (Fw: TCCTCCCTGTTCCAGAGACG; Rv: GGTCTCGCTCCTGGAAGATG).

Quantitative real-time PCR (qRT-PCR) was performed on CFX Opus 96 Real-Time PCR System (BIO-RAD, CA, USA) with a DyNAmo ColorFlash SYBR Green qPCR Kit (Thermo Fisher Scientific). Primers were synthesized by Eurofins Genomics Co., Ltd (Tokyo, Japan). BDNF (Fw: ACTGCAGTGGACATGTCTGG; Rv: CTGCAGCCTTCCTTGGTGTA); Tbp (Fw: AATGACTCCTATGACCCCTATCAC; Rv: AGGTCAAGTTTACAGCCAAGATTC).

### Western blotting

Myotubes were harvested by lysis buffer (containing 50 mM Tris–HCl pH 7.5, 1% NP-40, and other components), homogenized, and then centrifuged at 13,000×*g* for 15 min at 4 °C as described in our previous study [10]. The supernatant was collected and used for Western blotting. MyHC I (M8421, Sigma) and MyHC II (M4276, Sigma) were used as Type I and Type II fiber marker proteins respectively. BDNF (ab108319, Abcam, Cambridge, UK) was used to confirm BDNF protein expression in the TA. β-Actin (#4976, Cell Signaling Technology) was used as a housekeeping protein. Secondary antibodies used were as follows: rabbit or mouse horseradish peroxidase-conjugated secondary antibody (GE Healthcare, Buckinghamshire, UK.). The bands were quantified by ImageQuant TL Image Analysis software (Cytiva, MA, USA).

### Statistics

Data are expressed as means ± SEM (standard error of the mean). In this study, ‘N’ represents the number of mice, such that an ‘N’ value of 1 corresponds to the mean values obtained from several wells associated with an individual mouse. An unpaired Student’s *t*-test was performed to evaluate statistical differences between two groups. For multiple comparisons, data were analyzed using a one-way ANOVA, followed by Tukey post hoc tests. Values of p < 0.05 were considered to be statistically significant.

## Results

### Type I fibers express high levels of BDNF mRNA

**Fig. 1:**
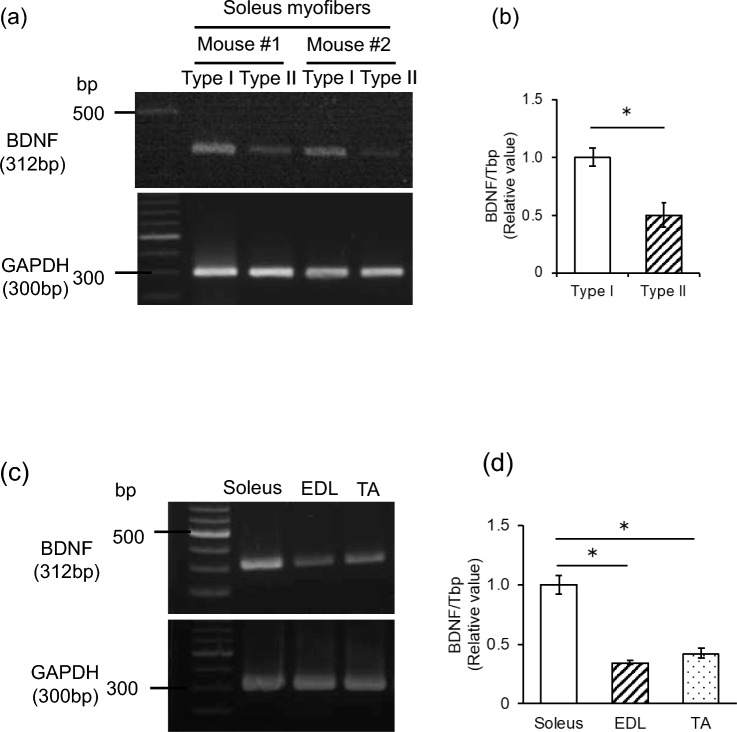
Expression of BDNF gene in Type I fiber and Type II fiber. **a** BDNF mRNA expression in Type I and II fibers. BDNF was detected by conventional RT-PCR and **b** BDNF mRNA expression level of Type I and Type II fibers was quantified by quantitative RT-PCR, N = 6, Data are shown as mean ± S.E.M., *; p < 0.05 by Student’s *t*-test. **c** BDNF mRNA expression in different muscle tissues detected by conventional RT-PCR and **d** BDNF mRNA expression level of different muscle tissues was quantified by quantitative RT-PCR. N = 6. Data are shown as mean ± S.E.M., *; p < 0.05 by one-way ANOVA followed by Tukey post hoc test

To investigate the BDNF gene expression levels in different muscle fiber types, we isolated Type I and Type II fibers separately from Myh7-CFP mice as described in the Methods. BDNF mRNA expression in Type I fiber is higher than Type II fiber in the soleus (Fig. 1a), being twice as elevated according to quantitative real-time PCR (Fig. 1b), in line with a recent study [12]. The expression of BDNF mRNA is higher in the soleus, a muscle where both Type I and II fibers coexist, compared to the EDL and TA which barely contain any Type I fibers (Fig. 1c). Quantitative real-time PCR results indicated that the expression level of BDNF mRNA in the soleus is approximately 2.5 times higher than the EDL and TA (Fig. 1d).

### BDNF doesn’t affect the proliferation and differentiation of myoblasts

**Fig. 2:**
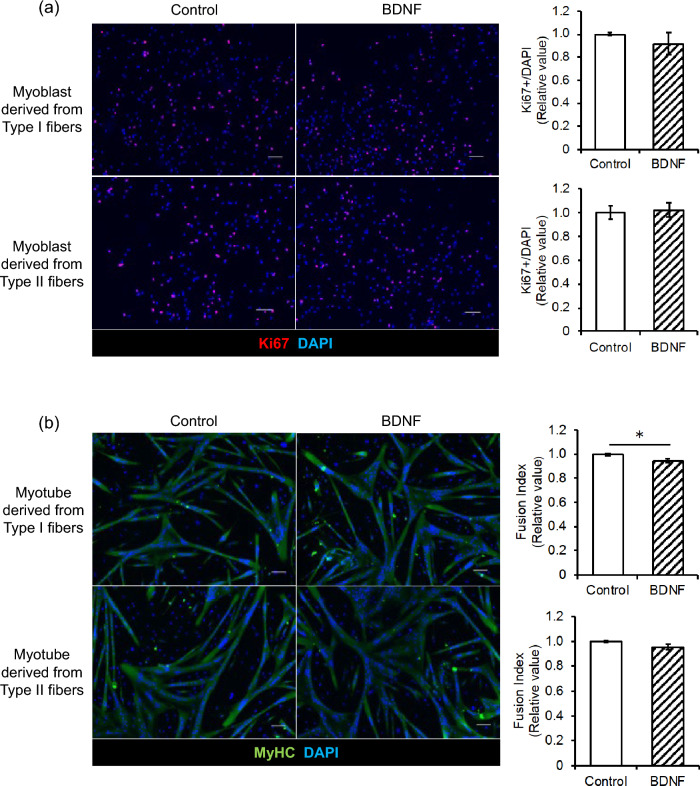
Myoblast proliferation and differentiation treated by BDNF. **a** The proliferating ratio of myoblasts was calculated by taking the number of Ki67 positive cells (red) divided by the total cell number (blue). **b** Fusion index of myotubes was calculated as the percentage of nuclei incorporated in the myotubes (green) relative to the total number of nuclei (blue). N = 2–3. Data are shown as mean ± S.E.M, *; p < 0.05 by Student’s *t*-test. Scale bar equal 100 µm. (Color figure online)

We examined the potential impact of BDNF on myoblast proliferation. After treating proliferating myoblasts with BDNF, the ratio of Ki67-positive cells, indicating proliferating cells, did not differ between BDNF treated myoblasts and untreated controls in either Type I or Type II-derived myoblasts (Fig. 2a). Subsequently, We examined the potential impact of BDNF on myoblast differentiation. After a 72-h BDNF treatment starting at the onset of differentiation, the fusion index, which measures myotube differentiation, was only reduced by 5% in BDNF treatment group compared to the control group (Fig. 2b). Although this aligns with previous findings, the small difference observed between the groups in this study suggests it is unlikely to significantly affect physiological responses [14].

### BDNF downregulates MyHC II expression in myotubes

**Fig. 3:**
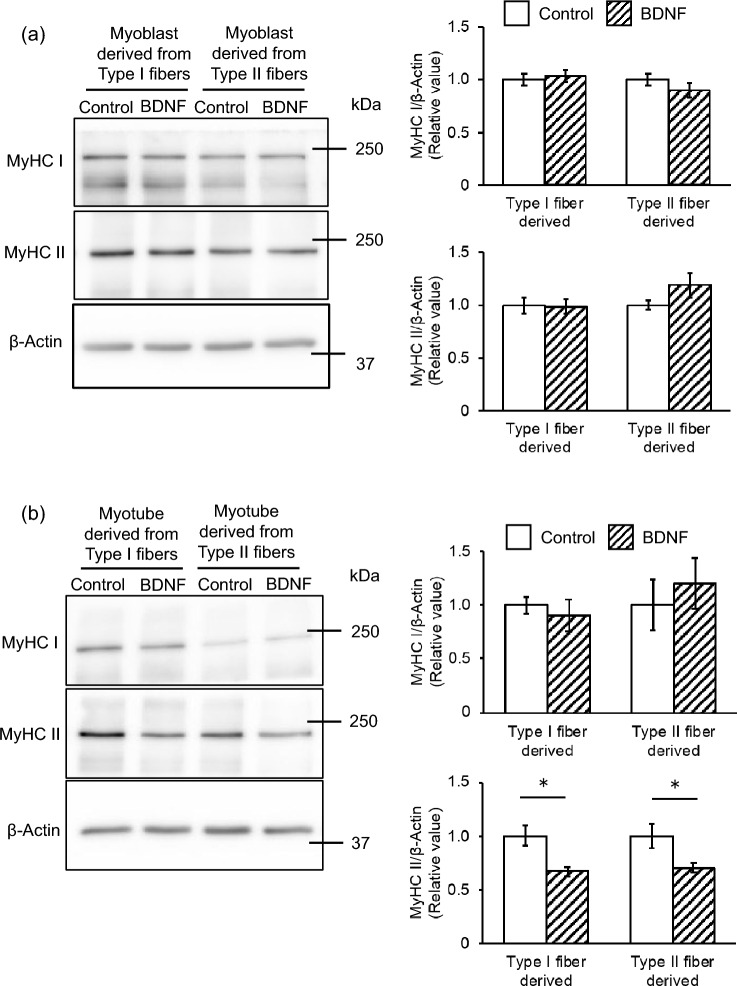
Effect of BDNF on fiber type determination during differentiation. **a** The expression levels of MyHC I and MyHC II proteins in the myotubes by 72 h BDNF treatment from the start or **b** from day 3 since the onset of differentiation were measured by western blotting. Representative images of immunoblotting were shown (left). The expression levels of these proteins were normalized to that of β-Actin (right). N = 8. Values are presented as mean ± S.E.M

To investigate the potential influence of BDNF on whether muscle cells differentiate into either Type I or Type II fibers, BDNF was supplemented in the differentiation media. Neither MyHC I nor MyHC II expression level was altered in either Type I or Type II-derived myotubes by 72 h BDNF treatment from the start of differentiation (Fig. 3a). However, MyHC II expression level was found to be decreased when the same treatment was applied from day 3 since the onset of differentiation in both Type I and Type II fiber-derived myotubes, while MyHC I was unaltered (Fig. 3b). We further confirmed the inhibitory effect of BDNF on MyHC II expression in myotubes is mediated by activating its high-affinity receptor TrkB [19]. MyHC II expression level is decreased by 72 h TrkB agonist PG003 treatment from day 3 (Supplement Fig. 1b), but not from start of differentiation (Supplement Fig. 1a). These findings suggest that BDNF downregulates the expression of MyHC II by activating TrkB in the late-stage differentiation of myotubes, which may result in the inhibition of the generation of Type II fibers.

### Overexpressing BDNF in regenerating TA inhibits Type IIA fiber generation

**Fig. 4:**
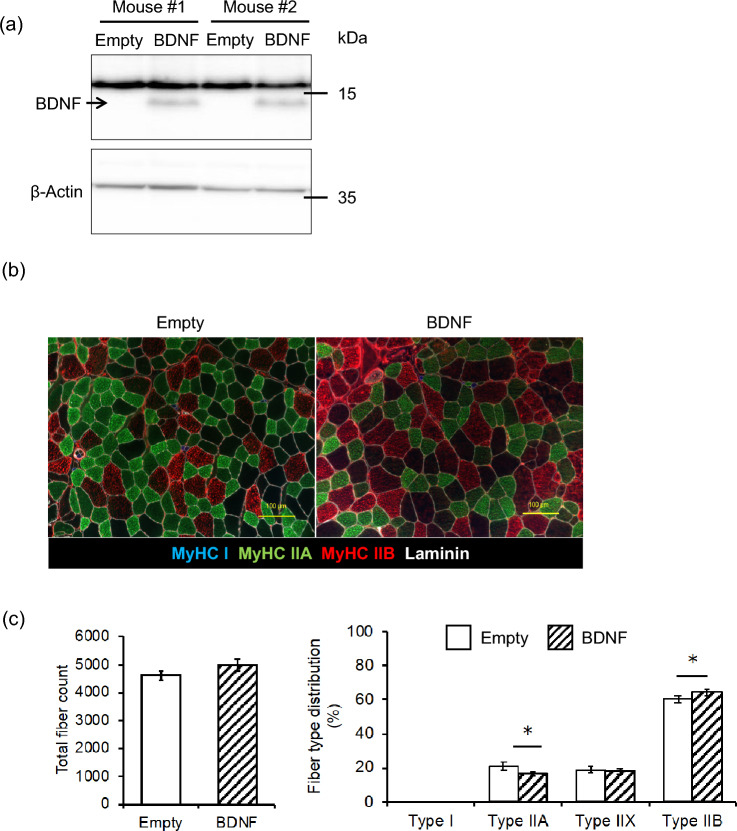
Fiber type change in regenerated TA by overexpressing BDNF. **a** Confirmation of BDNF overexpression in empty and BDNF vector-electroporated TA by Western blot. **b** Representative immunostaining images of regenerated TA. **c** Quantification of total fiber count and fiber type distribution of regenerated TA from immunostaining images, comparing the BDNF overexpressing group and the control group. N = 7, *; p < 0.05 by Student’s *t*-test. Values are presented as mean + S.E.M. Scale bar equal 100 µm

To confirm whether the downregulation of MyHC II expression by BDNF eventually affects Type II fiber generation, BDNF was overexpressed in the TA muscle 3 days after injury induced by an injection of cardiotoxin. TA were dissected and analyzed 14 days after the cardiotoxin injection. Western blotting was used to detect BDNF protein expression, recombinant BDNF was applied for a positive control. A clear band at 14 kDa, corresponding to mature BDNF, was observed in the BDNF overexpression group, while no detectable mature BDNF was found in the control group transfected with the empty vector (Fig. 4a). This observation is in agreement with a previous human study indicating the inability to detect mature BDNF with immunoblotting [12]. Bands between 25 and 37 kDa are likely to represent proBDNF, the precursor of mature BDNF (Supplementary Fig. 2), with a predicted molecular weight of approximately 28 kDa. Then we analyzed the cross section of regenerated TA (Fig. 4b). The total fiber count is comparable, but Type IIA fiber distribution is decreased in BDNF overexpression group (Fig. 4c). The distribution of Type IIB fibers increased in the BDNF overexpression group compared to empty vector control (Fig. 4c). This suggests that BDNF may inhibit Type IIA fiber determination during muscle regeneration in vivo.

## Discussion

Considering that BDNF is a well-known myokine [16] that is secreted by muscle cells [17], satellite cells situated proximally to muscle fibers could be affected by BDNF in a paracrine way. In the present study, we suggest that BDNF downregulates the expression of MyHC II in myotubes and this eventually leads to a decreased distribution of Type IIA fibers in regenerated muscle.

We first confirmed BDNF mRNA is highly expressed in Type I fibers. Our findings demonstrated that BDNF inhibits the expression of MyHC II in myotubes. Given that immature myotubes coexpress low levels of all MyHC II subtypes [18], and we cannot distinguish between the MyHC II subtype proteins (IIA, IIX and IIB) from cultured myotubes, it remains unclear whether BDNF specifically inhibits one or multiple MyHC II subtypes in myotubes.

Following muscle regeneration which leads to satellite cell activation and generation of new fibers, total fiber count was not altered by BDNF overexpression, indicating BDNF does not impede myofiber generation. The distribution of Type IIA fibers is diminished in regenerated muscle overexpressing BDNF, suggesting that BDNF specifically inhibits generation of Type IIA fibers in muscle tissue. This is consistent with the reduced expression level of MyHC II in BDNF treated myotubes, suggesting that BDNF may specifically downregulate the expression of MyHC IIA, consequently inhibiting Type IIA fiber generation.

We could not detect any Type I fiber in both control and overexpressing BDNF TA. Instead, the distribution of Type IIB fibers, defined as glycolytic fibers increased. This observation agrees with previous study that BDNF mediates glycolytic fiber-type specification [13]. We note that BDNF is one of many fiber type determination factors: when Type IIA fiber determination is inhibited by BDNF overexpression, other factors within TA may predominantly develop glycolytic and IIB types as a compensatory mechanism. The glycolytic property of the TA muscle explains the absence of oxidative Type I fibers in the BDNF overexpressing group.

The metabolic and myogenic properties of satellite cells vary depending on the fiber type from which they originate [20]. Therefore, we hypothesized that extrinsic factors, like BDNF, may exert different regulatory response in myotubes originating from Type I or II fibers. In the present study, BDNF downregulates MyHC II expression in myotubes derived from both fiber types similarly. These observations provide deeper insights into the physiological importance of elevated BDNF expression in the Type I fibers of the soleus, which is predominantly composed of Type I and IIA fibers. During the regeneration process following muscle injury, BDNF could be released from damaged Type I fibers, inhibiting myogenic cells from differentiating into Type IIA fibers, cooperating with other molecules to facilitate Type I fiber generation, thereby preserving fiber type distribution homeostasis during muscle regeneration.

A limitation of our study is that we did not overexpress BDNF in the soleus to confirm whether BDNF induces a shift from type IIA towards type I fibers. Future study could address this to further determine the role of BDNF in fiber type distribution. This could potentially resolve the discrepancy between Edman et al.’s [12] finding that BDNF is highly expressed in human Type I fibers (including our results) and Delezie et al.’s [13] finding that BDNF mediates Type IIB fiber specification.

## Conclusion

In conclusion, our work elucidates the physiological importance associated with the elevated expression level of BDNF within Type I fibers. BDNF downregulates Type II fiber-specific protein expression in differentiating myotubes and inhibits Type IIA fiber generation in muscle tissue, to preserve Type I fiber population and distribution in muscle tissue.

## Supplementary Information

Below is the link to the electronic supplementary material.

Supplementary Fig. 1 Effect of PG003 on myoblast and myotube. (a) The expression levels of MyHC I and MyHC II proteins in the myotubes by 72h PG003 treatment from the start or (b) from day 3 since the onset of differentiation were measured by Western blotting. Representative images of immunoblotting were shown (left). The expression levels of these proteins were normalized to that of β-Actin (right). N=5. Values are presented as mean±S.E.M, *; p<0.05 by Student’s t-test. Supplementary Fig. 2 Immunoblotting image of BDNF overexpressed TA and recombinant BDNF protein. Empty stands for a TA sample injected with an empty vector as a control. BDNF OE stands for BDNF overexpressed TA muscle. Rec BDNF stands for recombinant BDNF (2 ug) used as a positive control. Supplementary file1 (PPTX 286 KB)

## Data Availability

No datasets were generated or analyzed during the current study. All data generated or analyzed during this study are included in this published article.
