# Supplementary material for: Myokine BDNF highly expressed in Type I fibers inhibits the differentiation of myotubes into Type II fibers
Source: Mol Biol Rep. 2024 Nov 12;51(1):1143. doi: 10.1007/s11033-024-10044-3 (PMC11557626; doi:10.1007/s11033-024-10044-3)
Supplement: Supplementary file 1 — Supplementary Fig. 1 Effect of PG003 on myoblast and myotube. (a) The expression levels of MyHC I and MyHC II proteins in the myotubes by 72h PG003 treatment from the start or (b) from day 3 since the onset of differentiation were measured by Western blotting. Representative images of immunoblotting were shown (left). The expression levels of these proteins were normalized to that of β-Actin (right). N=5. Values are presented as mean±S.E.M, *; p<0.05 by Student’s t-test. Supplementary Fig. 2 Immunoblotting image of BDNF overexpressed TA and recombinant BDNF protein. Empty stands for a TA sample injected with an empty vector as a control. BDNF OE stands for BDNF overexpressed TA muscle. Rec BDNF stands for recombinant BDNF (2 ug) used as a positive control. Supplementary file1 (PPTX 286 KB) [file 11033_2024_10044_MOESM1_ESM.pptx]

## Slide 1
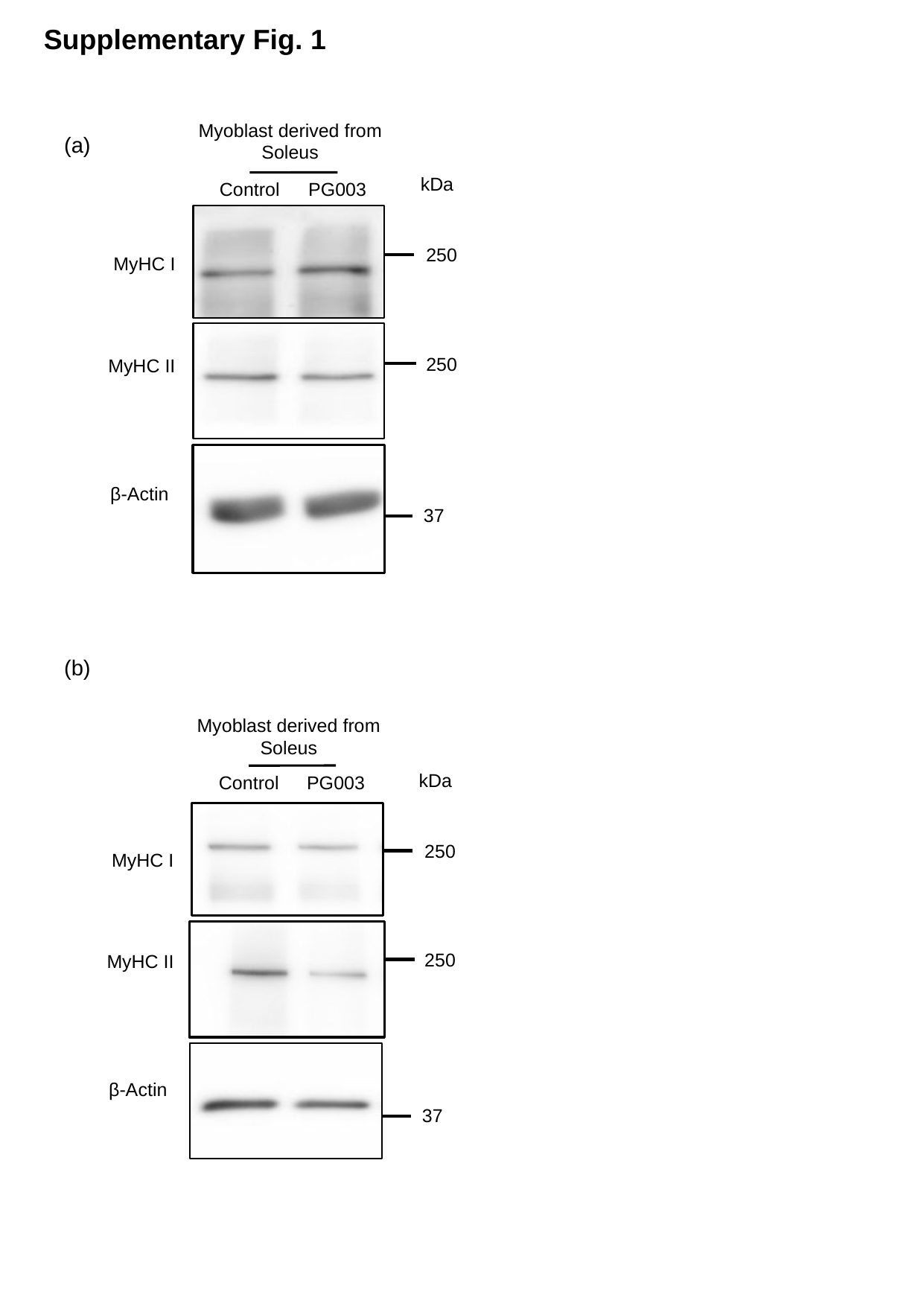

Supplementary Fig. 1
Myoblast derived from Soleus
(a)
kDa
PG003
Control
250
MyHC I
250
MyHC II
β-Actin
37
(b)
Myoblast derived from Soleus
kDa
PG003
Control
250
MyHC I
250
MyHC II
β-Actin
37

## Slide 2
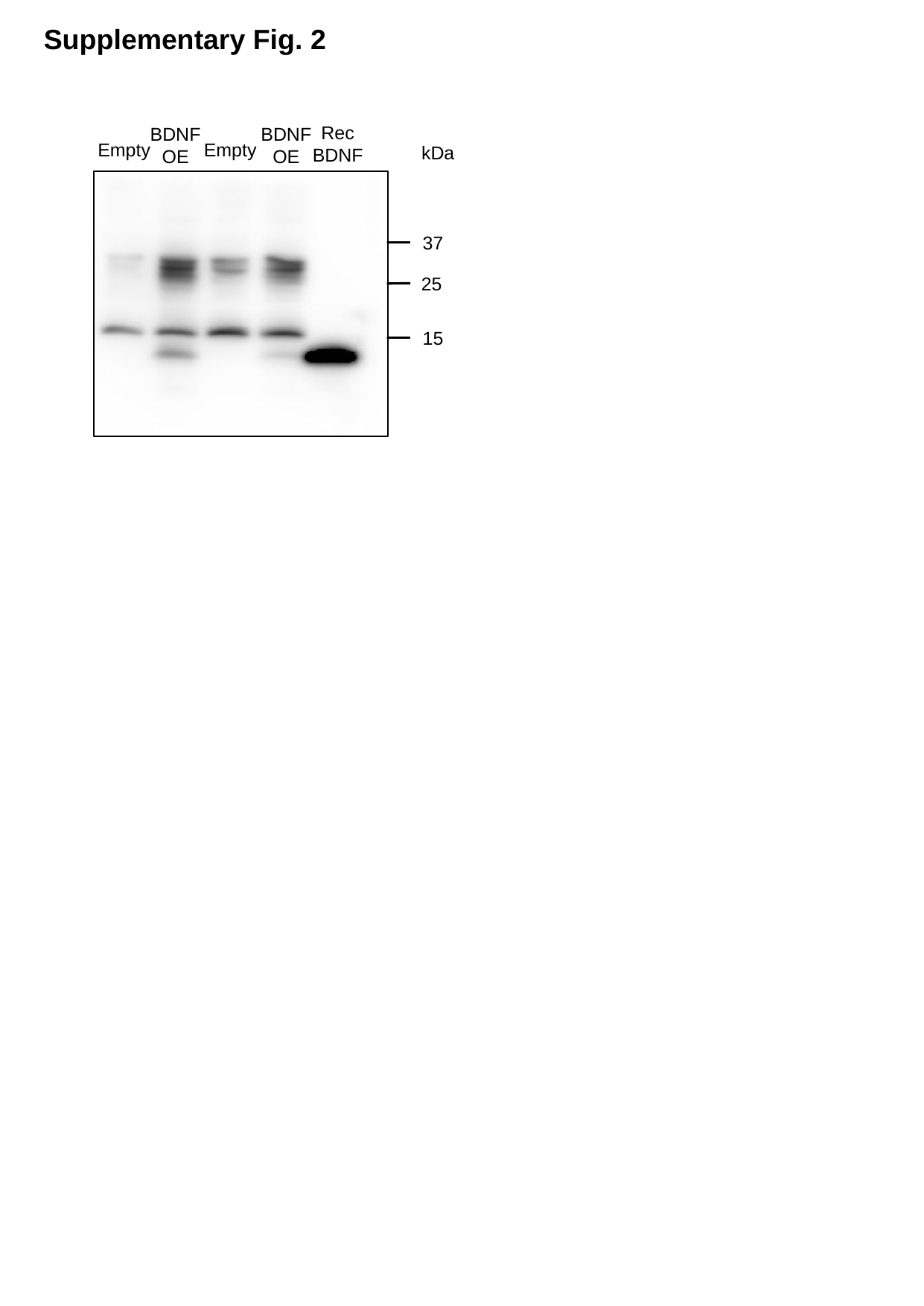

Supplementary Fig. 2
Rec
BDNF
BDNF
OE
BDNF
OE
Empty
Empty
kDa
37
25
15
